# 1.3 Micron Photodetectors Enabled by the SPARK Effect

**DOI:** 10.3390/mi16040440

**Published:** 2025-04-08

**Authors:** Teresa Crisci, Luigi Moretti, Mariano Gioffrè, Babak Hashemi, Mohamed Mammeri, Francesco Giuseppe Della Corte, Maurizio Casalino

**Affiliations:** 1 Department of Electrical Engineering and Information Technology, University of Naples Federico II, Via Claudio 21, 80125 Napoli, Italy; teresa.crisci@na.isasi.cnr.it (T.C.); mohamed.mammri@unina.it (M.M.); francescogiuseppe.dellacorte@unina.it (F.G.D.C.); 2 Institute of Applied Science and Intelligent Systems “Eduardo Caianiello” (CNR), Via P. Castellino n. 141, 800131 Naples, Italy; mariano.gioffre@cnr.it; 3 Department of Mathematics and Physics, University of Campania “Luigi Vanvitelli”, Viale Abramo Lincoln, 5, 81100 Caserta, Italy; luigi.moretti@unicampania.it; 4 DIIES Department, Mediterranea University of Reggio Calabria, 89124 Reggio Calabria, Italy; babak.hashemi@unirc.it

**Keywords:** waveguide, photodetectors, near-infrared, silicon, graphene

## Abstract

In this work, we present a graphene-based photodetector operating at a wavelength of 1310 nm. The device leverages the SPARK effect, which has previously been investigated only at 1550 nm. It features a hybrid waveguide structure comprising hydrogenated amorphous silicon, graphene, and crystalline silicon. Upon optical illumination, defect states release charge carriers into the graphene layer, modulating the thermionic current across the graphene/crystalline silicon Schottky junction. The photodetector demonstrates a peak responsivity of 0.3 A/W at 1310 nm, corresponding to a noise-equivalent power of 0.4 pW/Hz^1/2^. The experimental results provide deeper insights into the SPARK effect by enabling the determination of the efficiency × lifetime product of carriers at 1310 nm and its comparison with values previously reported at 1550 nm. The wavelength dependence of this product is analyzed and discussed. Additionally, the response times of the device are measured and evaluated. The silicon-based fabrication approach employed is versatile and does not rely on sub-micron lithography techniques. Notably, reducing the incident optical power enhances the responsivity, making this photodetector highly suitable for power monitoring applications in integrated photonic circuits.

## 1. Introduction

Silicon (Si) is widely acknowledged as a cornerstone material in semiconductor technology, having been extensively studied during the golden era of microelectronics [1,2,3]. However, its intrinsic properties, including its indirect bandgap, impose significant constraints on its efficiency in photonic applications, particularly for devices like lasers and near-infrared photodetectors (PDs) [4,5,6,7].

In recent years, graphene (Gr) and its two-dimensional counterparts have emerged as pivotal enablers of innovation in photonics and optoelectronics, thanks to their exceptional electronic, optical, and thermal properties [8,9,10,11]. Among the most promising applications of Gr is its integration into silicon-based PDs [12,13], particularly in waveguide-based architectures designed for near-infrared (NIR) detection [13,14,15]. These hybrid devices harness Gr unique characteristics for efficient conversion of light into electrical signals through several mechanisms.

One of the primary ways Gr PDs generate photocurrent is through the photovoltaic (PV) effect, where incident photons excite charge carriers at the Gr-Si interface, which are driven apart under the action of the built-in electric field, inducing a current flow through an external load [16]. This mechanism is particularly effective in high-speed applications due to the ultrafast carrier dynamics in Gr. However, its responsivity remains moderate, typically between 0.1 and 0.36 A/W at 1550 nm [17,18].

Beyond the photovoltaic response, Gr-based PDs can also exploit internal photoemission (IPE) [19,20], a process in which photons promote photoexcited carriers across an energy barrier into Gr, enhancing the PD response. This mechanism enables a modest but noticeable boost in responsivity, with values reaching approximately 0.37 A/W. The efficiency of IPE-based devices largely depends on the interface quality and the energy alignment between Gr and the underlying material.

Another crucial mechanism is the photo bolometric (PB) effect, which relies on Gr remarkable temperature sensitivity [21,22,23]. When incident light is absorbed, it locally heats the Gr layer, altering its electrical conductivity and leading to a measurable change in current. This effect is particularly valuable in scenarios where thermal fluctuations can be leveraged for enhanced detection, with devices achieving responsivity around 0.5 A/W [24].

A different yet highly promising approach is based on the photo thermoelectric (PTE) effect, which takes advantage of Gr ability to generate a voltage in response to a temperature gradient. Unlike the other mechanisms, PTE-based devices operate efficiently in voltage mode, where responsivity is defined as (*R_V_* = *V_ph_*/*P_inc_*) [25]. This approach has demonstrated remarkable results, with responsivity values ranging from 3.5 to 90 V/W at 1550 nm [26,27]. The ability to measure photovoltage directly without requiring an external bias makes PTE-based detectors highly attractive for energy-efficient and low-noise applications.

Multilayer structures composed of different materials play a crucial role in photonic applications, enabling enhanced light-matter interactions, tailored optical properties, and improved device performance [28,29,30,31]. By carefully engineering the composition and arrangement of these layers, researchers can achieve superior control over optical confinement, absorption, and carrier dynamics, which are essential for high-efficiency photodetection and modulation. A recent breakthrough in near-infrared light detection using Gr was achieved through the development of a hybrid waveguide structure composed of hydrogenated amorphous silicon (a-Si:H), Gr, and crystalline silicon (c-Si) [15,32]. By precisely embedding single layer Gr (SLG) within the waveguide (WG), the interaction between the guided optical mode and defect states at the SLG/a-Si:H interface is significantly enhanced [15]. Under NIR optical illumination, these defect states release charge carriers into the SLG layer, modulating the thermionic current across the SLG/c-Si Schottky junction. This phenomenon, referred to as SPARK (Schottky barrier Photo-modulation ARising from the Key role of traps), enabled a record responsivity of 1.9 A/W at 1550 nm [15].

The SPARK mechanism is thoroughly discussed in Ref. [15], where it is demonstrated that the responsivity of the device critically depends on the product *τ* × *η*. In this context, τ refers to the carrier lifetime, a measure of how long charge carriers can persist before recombination, while *η* represents the dimensionless conversion efficiency. This efficiency quantifies the fraction of charges trapped at the a-Si:H/SLG interface that are efficiently released into the SLG layer for each incident photon. The *τ* × *η* product serves as a pivotal factor in understanding the device performance, as it reflects the intricate interplay between photon interactions and trap dynamics within the material. Importantly, this relationship is not static but is strongly influenced by the wavelength of the incoming light. The dependence on wavelength suggests a complex, multifaceted mechanism where material properties, interface quality, and photon energy work together to govern charge behavior. Such insights are fundamental for optimizing device design and tailoring performance across different spectral ranges.

Although the SPARK effect has only been investigated at 1550 nm, another wavelength that could be explored is 1310 nm. Indeed, the most common single-mode optical fibers (e.g., ITU-T G.652 [33]) exhibit near-zero chromatic dispersion at 1310 nm, whereas dispersion at 1550 nm is significantly higher (~16–18 ps/(nm·km)) [34]. This increased dispersion at 1550 nm necessitates compensation techniques such as dispersion-compensating fiber (DCF) or advanced modulation formats, adding system complexity. Consequently, for short- to medium-range applications, including metro networks, local area networks (LANs), and data center interconnects, 1310 nm is often preferable because it mitigates the need for costly dispersion management. Furthermore, 1310 nm is also the preferred wavelength for multimode fiber systems, which are widely used in LANs and enterprise networks due to their lower-cost transceivers and compatibility with legacy infrastructure. The ability to use both single-mode and multimode fibers at 1310 nm provides additional flexibility, particularly in scenarios where cost and compatibility with existing infrastructure are key considerations [34].

In this paper, we investigate the behavior of the a-Si:H/SLG/c-Si WG photodetector enabled by the SPARK effect under 1310 nm illumination. Responsivity, noise-equivalent-power (NEP), and time response measurements have been performed. In this article, the experimental results provide a deeper understanding of the SPARK effect by determining the carrier efficiency/lifetime product at 1310 nm and comparing it with previously reported values at 1550 nm. The wavelength dependence of this product is analyzed and discussed, highlighting its implications.

## 2. SPARK Effect Theory

To ensure this work is self-contained, we briefly revisit the SPARK effect as described in Ref. [15]. SPARK has been observed in metal-semiconductor-metal (MSM) junctions, where one of the metals is Gr covered by a thin layer of a-Si:H. In principle, other amorphous materials could also be employed, as the key role is played by the traps localized at the interface between the amorphous material and Gr.

Under near-infrared (NIR) illumination, photons impart sufficient energy to trapped charge carriers, enabling them to overcome their binding potential and escape from localized trap states. Once freed, these carriers are subsequently transferred to the Gr layer, modifying its electronic properties. This charge injection process induces an upward shift in the Gr Fermi level, effectively reducing the Schottky barrier at the Gr/c-Si interface. The decrease in barrier height facilitates carrier transport across the junction, thereby enhancing the thermionic emission current. This barrier modulation, directly linked to trap-assisted charge dynamics, has been experimentally validated through precise thermionic current measurements, confirming the fundamental role of trap states in governing the device optoelectronic response [15]. The current emitted through the Gr/c-Si Schottky barrier is collected by the second electrode on the c-Si made of aluminum (Al).

**Figure 1:**
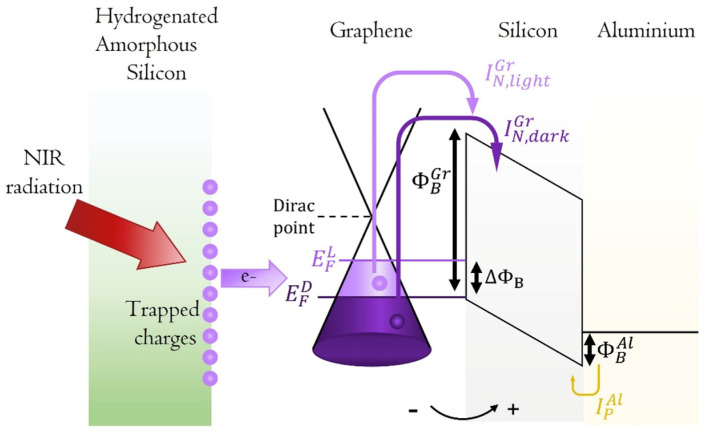
Band diagram of the Gr/c-Si/Al metal semiconductor metal junction in flat-band conditions [35]. The Gr is capped with the a-Si:H. Under NIR light irradiating the trap states at the a-Si:H/Gr interface, electrons are released in the Gr increasing the Fermi level from $E_{F}^{D}$ (Fermi level in dark) to $E_{F}^{L}$ (Fermi level upon illumination). Consequently, a higher thermionic current $I_{N,light}^{Gr}$ flows in the device because of the smaller Gr/c-Si Schottky barrier $\phi_{B}^{Gr}$ [15,32]. ${\Delta \phi }_{B}$ represents the Fermi level shift upon NIR illumination while $I_{P}^{Al}$ the hole current flowing through the Al/c-Si Schottky junction $\phi_{B}^{Al}$.

The detection mechanism is illustrated in the band diagram in Figure 1, which depicts the behavior of the Gr/c-Si/Al junction under a voltage exceeding the flat-band voltage, where the conduction and valence bands of silicon can be considered nearly linearized [35].

When a negative voltage is applied to the Gr electrode in relation to the grounded Al electrode, the Gr/p-Si junction is forward-biased, while the Al/p-Si junction is reverse-biased, leading to the energy band diagram shown in Figure 1 under dark conditions. As depicted in Figure 1, the current passing through the device prior to illumination (*I_TD_*) is the total of the dark current, which is caused by thermionic emission of electrons overcoming the potential barrier $\phi_{B}^{Gr}$ at th e Gr contact ($I_{N,\;dark}^{Gr}$), and the thermionic emission of holes, which overcome the potential barrier $\phi_{B}^{Al}$ at the Al contact (*I_P_^Al^*).

(1) $$I_{TD}={I_{N,\;dark}^{Gr}+I}_{P}^{Al}={A_{Gr}A_{N}^{*}T^{2}e^{-\frac{{q\phi }_{B}^{Gr}}{kT}}+A}_{Al}A_{P}^{*}T^{2}e^{-\frac{{q\phi }_{B}^{Al}}{kT}}$$

where *A_Gr_* and *A_Al_* represent the areas of Gr and Al in contact with c-Si respectively, *T* is the absolute temperature, *k* is the Boltzmann constant, and A*_P_ and A*_N_ are the Richardson constants for P-type and N-type silicon, respectively. As discussed in Ref. [32], the principle of current continuity requires that the total current in the MSM structure be equal to the sum of the thermionic currents passing through both junctions.

Upon NIR illumination of the Gr active region, the observed increase in the total current flowing through the device (*I_TL_*) indicates a reduction in the Schottky barrier ∆*ϕ_B_* at the Gr/c-S interface [32].

(2) $$I_{TL}={I_{N,\;light}^{Gr}+I}_{P}^{Al}=A_{Gr}A_{N}^{*}T^{2}e^{-\frac{q\left(\phi_{B}^{Gr}-∆\phi_{B}\right)}{kT}}+A_{Al}A_{P}^{*}T^{2}e^{-\frac{q\phi_{B}^{Al}}{kT}}$$

If the photogenerated current is defined as *I_ph_* = *I_TL_* − *I_TD_*, we can write:

(3) $$I_{Ph}=A_{Gr}A_{N}^{*}T^{2}e^{-\frac{{q\phi }_{B}^{Gr}}{kT}}\left(e^{\frac{q∆\phi_{B}}{kT}}-1\right)$$

On the other hands, the change in Schottky barrier ${\Delta \phi }_{B}$ under illumination can be defined as [32]:

(4) $$q∆\phi_{B}={∆E}_{F}^{L}-{∆E}_{F}^{D}=-sign\left(n_{0}+N_{c}\right)\hbar v_{F}\sqrt{\pi \left|n_{0}+N_{c}\right|}+sign\left(n_{0}\right)\hbar v_{F}\sqrt{\pi \left|n_{0}\right|}$$

where $∆E_{F}^{D}=E_{F}^{D}-E_{FO}$ and $∆E_{F}^{L}=E_{F}^{L}-E_{FO}$ represent the differences between the Fermi level and the Dirac point $E_{FO}$ under both dark and illuminated conditions, *k_B_* is the Boltzmann constant, ℏ is the Plank constant, *v_F_* the Fermi velocity and *n*_0_ is the capped Gr doping [36]. It is interesting to note that the mathematical operator sign in Equation (4) takes different values depending on the doping of graphene. In the case of P-type doping, graphene has an excess of positive charges (holes), so the sign operator takes a positive value (+). Conversely, with N-type doping, graphene has an excess of negative charges (electrons), and the sign operator takes a negative value (−). The parameter *N_c_* refers to the charge carriers trapped at the a-Si:H/Gr interface, which are released into Gr upon illumination. The value of *N_c_* is solely determined by the rate at which charges are generated and transferred from the a-Si:H interface to Gr. Defining the photon flux as $\psi =\frac{P}{\left(\hbar \omega \cdot A_{Gr}\right)}$, where *P* is the incident optical power (in eV/s) able to illuminate the whole *A_Gr_* active area of the a-Si:H/Gr interface (in cm^2^), and ℏω is the photon energy, *N_c_* can be related to the photon flux using the following expression [15,32]:

(5) $$N_{c}=\tau \cdot \eta \cdot \frac{P}{\left(\hbar \omega \;\cdot A_{Gr}\right)}$$

where *τ* can be interpreted as the carrier lifetime, representing the average duration for which a trap remains occupied by a charge, while *η* denotes the conversion efficiency (dimensionless), which is the number of charges trapped at the a-Si:H/Gr interface that are released into Gr per incident photon. The conversion efficiency is influenced by the interaction between photons and traps. Considering Equations (3)–(5), we can express the relationship as:

(6) $$R=\frac{I_{Ph}}{P}=\frac{A_{Gr}A_{N}^{*}T^{2}e^{-\frac{{q\phi }_{B}^{Gr}}{kT}}}{P}\left[e^{\frac{\hbar v_{F}}{kT}\cdot \left(-\sqrt{\pi \left|n_{0}-\tau \cdot \eta (P)\cdot \frac{P}{\hbar \omega \;\cdot \;A_{Gr}}\right|}+\sqrt{\pi \left|n_{0}\right|}\right)}-1\right]$$

This equation assumes that the transferred Gr inherently exhibits P-type doping, and that no inversion of doping occurs in Gr under NIR illumination (|N_c_| < |n_0_|). Finally, by modelling the efficiency-lifetime carrier product as the power function τη (P) = α/P^β^ [32] and under the assumption |N_c_| << |n_0_|, we obtain:

(7) $$R≅\left[A_{N}^{*}T^{2}e^{-\frac{{q\phi }_{B}^{Gr}}{kT}}\cdot \left(\frac{\pi }{2}\cdot \frac{\hbar v_{F}}{kT}\cdot \frac{\alpha }{\sqrt{\pi n_{o}}\cdot h\nu }\right)\right]\cdot P^{-\beta }$$

where *R* can also be interpreted as the SPARK effect photo-gain, which is determined by the change in thermionic current resulting from the photo-induced modulation of the Gr Fermi level, facilitated by interfacial traps. Equation (7) clearly illustrates that the responsivity *R* decreases as the optical power P increases, and that R is strongly influenced by the Gr Schottky barrier ${q\phi }_{B}^{Gr}$.

## 3. Device Concept and Fabrication

**Figure 2:**
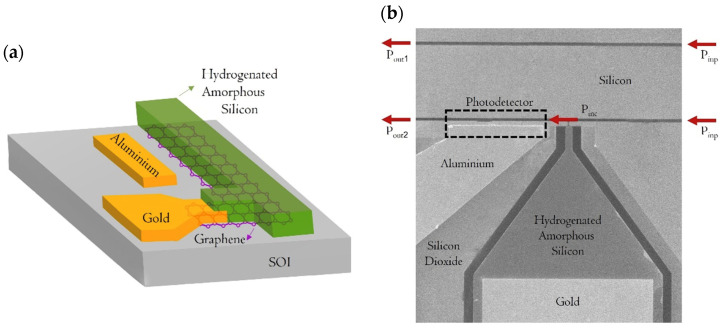
(**a**) Sketch of the Gr-based PD integrated into the hybrid c-Si/a-Si:H WG. (**b**) SEM image of the fabricated device illustrating the measurements of the incident optical power on the Gr Layer $P_{inc}$. At the top of the image, there is the c-Si/a-Si:H waveguide used for the measurements of the incident optical power $P_{out1}$. The other waveguide includes the SLG layer located between c-Si and a-Si:H, highlighted within the black dashed line.

The suggested PD utilizes a hybrid WG structure, integrating a-Si:H, SLG, and c-Si components, all precisely fabricated on a Silicon-On-Insulator (SOI) platform. The configuration employs a rib waveguide design, embedding SLG within the core, which consists of a-Si:H and c-Si, as depicted in Figure 2a. This deliberate design focuses the optical mode at the SLG/a-Si:H interface, where a significant density of charge carriers becomes trapped [15].

Figure 2a offers a detailed visualization of the active device, highlighting the aluminum (Al) electrode and the SLG positioned on c-Si. The junction formed by SLG/c-Si/Al represents a metal-semiconductor-metal (MSM) structure [35], with its associated energy band diagram illustrated in Figure 1. The a-Si:H layer plays a crucial role in this system due to its refractive index being closely aligned with that of c-Si, effectively minimizing optical discontinuities for the propagating infrared light.

The production process was carefully planned to ensure compatibility with CMOS technology, employing low thermal-budget techniques aligned with the Back-End-of-Line (BEOL) requirements. To safeguard the structural integrity of the Gr, high-temperature treatments were intentionally scheduled at the beginning of the workflow. The production process has been thoroughly described in Ref. [15] and is briefly referenced here for the reader’s convenience.

The procedure began with an RCA cleaning protocol, which effectively removed contaminants from the SOI substrate to prevent potential defects in the final device.

Next, a thermal oxidation step was performed at 1100 °C, a temperature that could potentially damage materials introduced in later stages, such as SLG and metallic contacts. Conducted in a controlled nitrogen atmosphere, this four-hour process yielded a 50 nm-thick silicon dioxide (SiO_2_) layer. Selective etching of SiO_2_ was subsequently performed using a Buffer Oxide Etch (BOE) solution, to realize the aluminum (Al) electrode. This step involves photolithography, aluminum (Al) thermal evaporation, and a lift-off process. To enhance adhesion between the metal and the substrate, thermal annealing was performed at 475 °C for 30 min.

Next, 1 cm × 1 cm SLG, sourced from Graphenea Inc. (San Sebastian, Spain), was transferred onto the chip using the procedure described in Ref. [37]. Through a photolithography process, SLG was protected by a photoresist before being shaped into a 200 µm-long strip using O_2_ plasma dry etching.

Electrical contacts with SLG were established through another photolithography step, followed by the thermal evaporation of Cr/Au (7/100 nm). The chromium (Cr) layer ensured strong adhesion between gold (Au) and the substrate, while a bilayer photoresist system facilitated a smooth lift-off process to prevent damage to the SLG.

The final phase involved defining the WGs. A 110 nm-thick a-Si:H layer was deposited at 100 °C using Plasma-Enhanced Chemical Vapor Deposition (PECVD). Photolithography and selective dry etching with a CF_4_ and O_2_ gas mixture were then used to pattern the rib waveguide. Lastly, the chip was cleaved, producing a 6.5 mm-long waveguide, as depicted in the SEM images displayed in Figure 2b.

A Raman analysis of the graphene quality after the deposition of a-Si:H was performed in Ref. [15], demonstrating that graphene retains its characteristics unchanged after the deposition process.

## 4. Experimental Results and Discussion

### 4.1. Electrical Characterization

**Figure 3:**
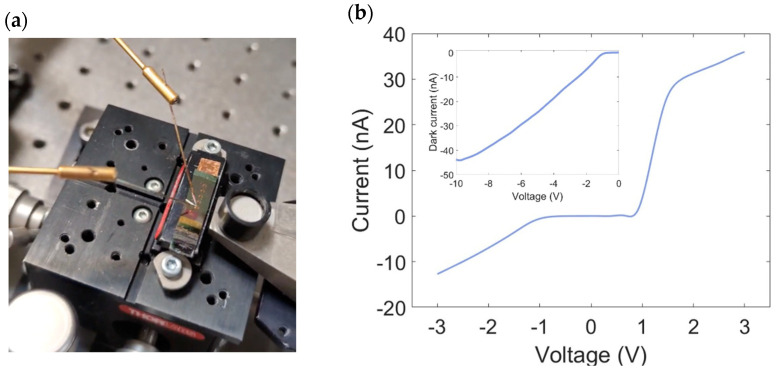
(**a**) Photo of the device during electro-optical measurements, showing two probes biasing the PD on the right and the optical fiber aligned with the PD waveguide on the left. (**b**) IV characteristics of the Gr/c-Si/Al MSM junction in dark conditions. The inset provides a more detailed view over a wider range, focusing on the region where c-Si is grounded and Gr is subjected to a negative bias, representing the typical operating conditions of our photodetector.

The electrical performance of the devices was evaluated using a probe station paired with a source meter (Keithley Instrument Inc., Cleveland, OH, USA). Electrical contact was achieved using two precision metal probes, which were carefully placed on the device mounted on the probe station holder, as displayed in Figure 3a. These probes were attached to micromanipulators, securely fixed to the optical table via a vacuum system.

By varying the voltage applied across the electrodes, the resulting current flowing through the device was recorded, enabling analysis of the structure’s electrical properties and extraction of the I-V characteristics. Each I-V curve represents the average of three consecutive measurements.

The current-voltage (I-V) characteristics clearly demonstrated the Metal-Semiconductor-Metal (MSM) behavior of the Gr/c-Si/Al junction, as shown in Figure 3b. This structure comprises two distinct Schottky interfaces: Gr/c-Si and Al/c-Si. When an external voltage was applied across the device, one junction operated under forward bias while the other was in reverse bias. A key parameter in this configuration is the flat-band voltage [35], which depends on factors such as the electron charge, the doping concentration of the semiconductor, the dielectric constant of silicon, and the separation between the two electrodes. At this specific voltage, the c-Si layer becomes fully depleted, causing the energy bands at the reverse-biased junction to flatten.

### 4.2. Responsivity Measurements

To evaluate the PD responsivity, it is essential to accurately measure the optical power incident on the active region of the SLG, denoted as *P_inc_*. It is important to highlight that the Gr absorption layer, measuring 200 μm in length, was integrated into a 6 mm-long optical waveguide.

The incident optical power was determined using a reference waveguide, fabricated specifically for this purpose and placed adjacent to the one containing the PD as shown in Figure 2b. First, we measured the optical power output (*P_out_*_1_) from the reference waveguide after coupling a known input optical power (*P_inp_*), which was measured using a tapered optical fiber aligned with a commercial, calibrated free-space InGaAs photodiode. To clarify the relationship between *P_inp_* and *P_inc_*, we can state that *P_inp_* represents the optical power in air emitted from the tapered optical fiber, while *P_inc_* corresponds to the remaining optical power at the photodetector input due to coupling and propagation losses. Next, the measurement was repeated with the waveguide containing the PD, yielding an output power value (*P_out2_*). Under the assumption that both guides are characterized by the same coupling and propagation losses, the difference between these two measurements (*P_abs_* = *P_out1_* − *P_out2_*) represents the optical power absorbed by the SLG.

**Figure 4:**
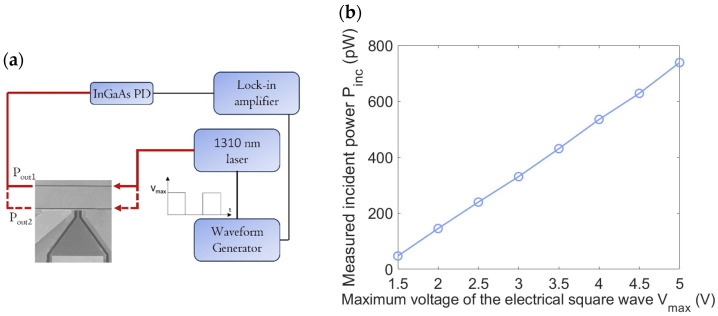
(**a**) Schematic of the experimental setup for measuring incident optical power. (**b**) Incident optical power values on the Gr layer embedded between a-Si:H and c-Si, measured at varying peak voltages (*V_max_*) of the electrical square wave, ranging from 1.5 V to 5 V.

The experimental setup, illustrated in Figure 4a, is relatively straightforward. A continuous-wave 1310 nm laser source (Thorlabs, Milan, Italy) is modulated at 270 Hz using an external waveform generator producing an electrical square wave. Light is coupled into the waveguide via a tapered optical fiber, while the output light is collected by a multimode optical fiber with a core diameter of approximately 70 μm. The collected light is then converted into an electrical current by a calibrated InGaAs PD, and is subsequently analyzed using a lock-in amplifier.

Our experimental results indicate that *P_out_*_2_ is close to zero. This suggests that nearly all the incident optical power is absorbed by the Gr strip, allowing us to approximate *P_inc_* as being nearly equal to *P_abs_* ≈ *P_out_*_1_.

Measurements were performed by varying the maximum voltage of the electrical square wave (*V_max_*) from 1.5 V to 5 V and recording the corresponding *P_inc_* values. The results are presented in Figure 4b.

**Figure 5:**
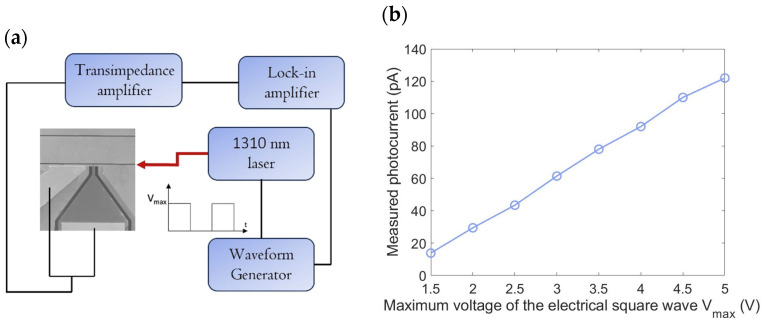
(**a**) Schematic of the experimental setup for measuring photogenerated current. (**b**) Measured photogenerated current values on the Gr layer embedded between a-Si:H and c-Si, measured at different peak voltages (*V_max_*) of the electrical square wave in a range from 1.5 V to 5 V.

To measure the photocurrent generated by the PD under test, a similar experimental setup was employed. While the input optical configuration remains unchanged, the output signal is now an electrical current extracted using two precision metal probes. These probes are connected to a transimpedance amplifier, which converts the photocurrent into a voltage signal. The transimpedance amplifier is also able to apply a bias of −10 V to the PD. The resulting voltage is then measured using a lock-in amplifier for accurate analysis. The experimental setup and corresponding results are presented in Figure 5a and 5b, respectively.

**Figure 6:**
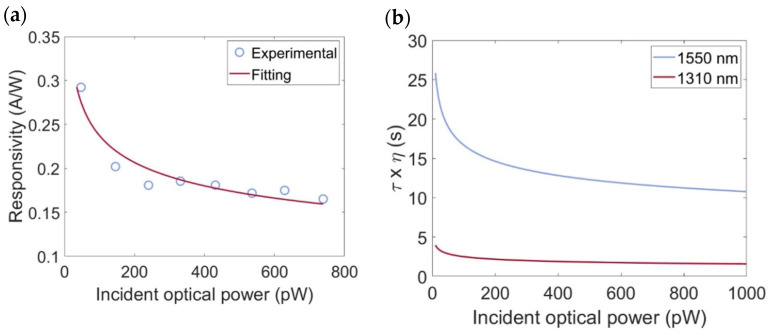
(**a**) Responsivity of the SPARK effect-based PD at 1310 nm as a function of incident optical power, with the corresponding curve fit (red) based on Equation (6). (**b**) Dependence of the efficiency-lifetime carrier product on incident optical power at 1310 nm (red curve) and 1550 nm (blue curve).

From the data shown in Figure 4b and Figure 5b, it is possible to derive the responsivity which exhibits a strong dependence on the incident optical power as shown in Figure 6a.

Notably, the PD demonstrated a maximum responsivity of 0.3 A/W at a wavelength of 1310 nm and a bias voltage of −10 V, corresponding to an incident optical power of approximately 47 pW. As the incident optical power increases, the responsivity gradually decreases and eventually stabilizes at approximately 0.17 A/W.

To further interpret these results, we fitted the experimental data presented in Figure 6a using Equation (6) described in Section 2. During the optimization process, the following parameters were employed: *A_Gr_* = 1.4 × 10^−5^ cm^2^, $A_{N}^{*}\;$= 112 A/cm^2^ K^2^, T = 300 K, *k_B_* = 8.61 × 10^−5^ eV/K, ℏ = 6.58 · 10^−16^ eVs, $v_{F}=1.1\;\cdot \;10^{8}\;\mathrm{cm}/s$, hυ=0.95 eV, $q\phi_{B}^{Gr}=0.56\;\mathrm{eV}$ [15], and *n*_0_ = 0.24 × 10^12^ cm^−3^ [15].

The parameter τη (P_inc_) was determined through the fitting procedure. Referring to [15], the carrier efficiency-lifetime product can be described as a power-law function τη (P_inc_) = α/P^β^_inc_. The fitting process produced values of α = 0.025 × 10^−6^ sW^β^ and β = 0.2, with an R-square of 91%, indicating good agreement between the experimental data and the model (Figure 6a).

To further analyze the results, a comparison was made between the efficiency-lifetime carrier product at a wavelength of 1310 nm, as reported in this study, and that at 1550 nm, documented in [15] as shown in Figure 6b. For 1310 nm, we obtained *τη* (*P_inc_*) = (0.025 × 10^−6^)/(*P_inc_*)^0.2^ (s), while at 1550 nm, the expression was *τη* (*P_inc_*) = (0.21 × 10^−6^)/(*P_inc_*)^0.19^ (s). Notably, the parameter α exhibited a marked dependence on wavelength, while β remained nearly constant.

The dependence of the efficiency-lifetime carrier product τη on incident optical power at both wavelengths is illustrated in Figure 6b. By using Equation (7), it was demonstrated that the SPARK effect enhances gain by a factor of 8 at 1550 nm relative to 1310 nm. This strong wavelength dependence originates from a combination of factors, including the electronic properties of a-Si:H and the quality of both a-Si:H and the a-Si:H/SLG interface. It is well-known that a-Si:H is characterized by a high density of localized states within the bandgap due to structural disorder and the presence of dangling bonds. These states act as carrier traps, capturing electrons or holes and significantly affecting their lifetime τ, and the efficiency of charge extraction η is governed by the ability of these trapped carriers to be released and subsequently transferred to Gr. The process of carrier release is influenced by both thermal excitation and optical excitation, which depend strongly on the energy distribution of trap states and the energy of the incident photons. At 1310 nm, the incident photons have a higher energy (~0.95 eV) compared to 1550 nm (~0.8 eV). It can be speculated here that this difference in photon energy may be responsible for the reduction in the overall charge transferred to SLG and may limit the τη product achieved at 1310 nm. The presence of higher-energy photons may enable charges to reach trap states with shorter lifetimes. Moreover, since at 1310 nm it can be assumed that deeper trap states are involved, it is possible that some re-trapping mechanisms occur, and that they are responsible for the reduction in the τη product. Naturally, these considerations should be validated by experimental measurements on the materials in question, which we believe falls outside the scope of this manuscript.

The sensitivity of the system was evaluated using the noise equivalent power (NEP), which quantifies the optical power needed, per square root of bandwidth, to produce a photocurrent equivalent to the noise contribution. NEP was calculated as NEP = *i_n_*/*R*, where *R* = 0.3 A/W at 47 pW, the dark current *I_d_* = 45 nA at −10 V (inset of Figure 3b), and $i_{n}=\sqrt{2qI_{d}}$. This yielded an NEP of 0.4 pWHz^−1/2^.

### 4.3. Time Response

**Figure 7:**
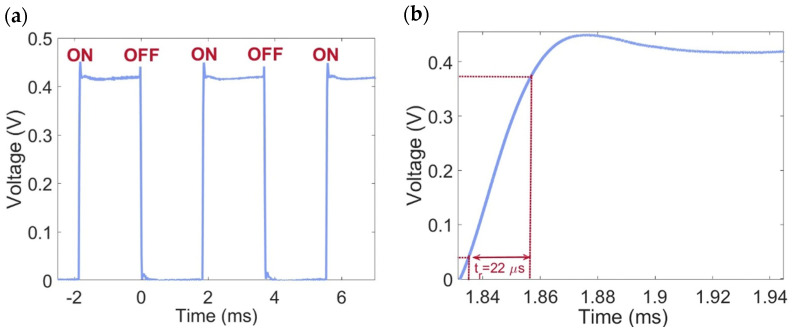
(**a**) Real-time voltage generation of the photodiode (PD) under 1310 nm-light exposure. (**b**) Rising part of the real-time photocurrent response, showing a rise time of 22 μs.

The real-time photocurrent response of the PD is depicted in Figure 7a. The time-dependent photocurrent of the PD was measured by periodically toggling a near-infrared laser on and off while applying a −10 V reverse bias, as shown in Figure 7a. Upon laser activation, the photocurrent rises to a saturation level and drops to a baseline (dark current) when the laser is turned off.

As illustrated in Figure 7b, where the rising part of the real-time photocurrent response is reported, a rise time (*t_r_*) of 22 μs can be observed. The t_r_ is defined as the duration required for the photocurrent to escalate from 10% to 90% of its saturation value, and it is related to the angular frequency by the equation *ω*_3dB_ = 2π(0.35/*t_r_*). Consequently, a 3 dB angular frequency of approximately 0.1 MHz can be estimated. The limited response speed is attributed to the significant influence of trap states in the detection mechanism. The prolonged carrier lifetime (*τ*) typically impacts the device speed, a phenomenon previously observed in Gr PDs that utilize the photogating effect, where a balance must be struck between bandwidth and gain. Carrier lifetime is closely associated with the energy band structure, as well as with the defects and impurities, which are heavily dependent on the quality of the a-Si:H and the deposition process parameters. Therefore, meticulous defect engineering would be essential to optimize the efficiency-bandwidth product.

Our SPARK-based PDs exhibit high sensitivity, making them particularly suitable for power monitoring applications where high speeds are not required [38,39,40]. Within photonic integrated circuits (PICs), these detectors play a crucial role in measuring optical power levels. They operate by extracting minimal optical power at specific points in the circuit, ensuring the proper functioning of the system. On-chip power monitoring and calibration are essential for various tasks, such as tuning resonance frequencies in sensing circuits or compensating for phase errors in optical phased arrays (OPAs).

## 5. Conclusions

In this work, we have investigated a WG PD under 1310 nm illumination, leveraging the SPARK effect within a hybrid waveguide structure composed of a-Si:H, SLG, and c-Si.

The experimental results have been summarized in Table 1 and compared with those previously obtained at 1550 nm [15].

The device operating at 1310 nm exhibits a peak responsivity of 0.3 A/W and a noise-equivalent power of 0.4 pW/Hz^1^/^2^, demonstrating lower efficiency but higher sensitivity compared to its counterpart at 1550 nm.

Moreover, for the first time, the device’s time response associated with the SPARK effect has been measured. The PD exhibits a limited response speed, with a rise time of 22 μs corresponding to a 3 dB angular frequency of approximately 0.1 MHz. This time response is governed by trap states in the detection mechanism, highlighting the trade-off between bandwidth and gain typical of Gr-based PDs utilizing the photogating effect.

Finally, our experimental results provided valuable insights into the SPARK effect by determining that the efficiency × lifetime product exhibited a marked dependence on wavelength. It was demonstrated that the SPARK effect enhances photo-gain R by a factor of 8 at 1550 nm relative to 1310 nm.

Therefore, while the response speed is limited, the high sensitivity of these PDs makes them ideal for power monitoring applications in photonic integrated circuits (PICs), where precise on-chip power measurements are necessary for tasks such as resonance frequency identification, system calibration, and phase correction in optical phased arrays (OPAs).

## Figures and Tables

**Table 1:** Performance of the 1310 nm PD described in this work compared to the 1550 nm PD reported in [15].

Wavelength	Responsivity	NEP	Rise Time	Efficiency-Lifetime Product τ × η (P_inc_) = α/(P_inc_)^β^
1310 nm	0.3 A/W	0.4 pWHz^−1/2^	22 μs	α = 0.025 × 10^−6^ sW^β^ β = 0.2
1550 nm [15]	1.9 A/W	9.6 pWHz^−1/2^	-	α = 0.21 × 10^−6^ sW^β^ β = 0.19

## Data Availability

The datasets generated and analyzed during the current study are available from the corresponding author upon reasonable request.
